# Novel anti-HER2 ADCs vs dual anti-HER2 antibody for HER2-positive metastatic breast cancer failed to tyrosine kinase inhibitor

**DOI:** 10.1093/oncolo/oyae144

**Published:** 2024-12-31

**Authors:** Feng Li, Jianbin Li, Chenchen Ji, Song Wu, Shaohua Zhang, Tao Wang, Li Bian, Zefei Jiang

**Affiliations:** Department of Breast Oncology, The Fifth Medical Center of Chinese PLA General Hospital, Beijing 100071, People’s Republic of China; Department of Breast Oncology, The Fifth Medical Center of Chinese PLA General Hospital, Beijing 100071, People’s Republic of China; Department of Medical Molecular Biology, Beijing Institute of Biotechnology, Academy of Military Medical Sciences, Beijing 100071, People’s Republic of China; Department of Breast Oncology, The Fifth Medical Center of Chinese PLA General Hospital, Beijing 100071, People’s Republic of China; Department of Physical Examination, Beijing Electric Power Hospital, Beijing 100000, People’s Republic of China; Department of Breast Oncology, The Fifth Medical Center of Chinese PLA General Hospital, Beijing 100071, People’s Republic of China; Department of Breast Oncology, The Fifth Medical Center of Chinese PLA General Hospital, Beijing 100071, People’s Republic of China; Department of Breast Oncology, The Fifth Medical Center of Chinese PLA General Hospital, Beijing 100071, People’s Republic of China; Department of Breast Oncology, The Fifth Medical Center of Chinese PLA General Hospital, Beijing 100071, People’s Republic of China; Department of Breast Oncology, The Fifth Medical Center of Chinese PLA General Hospital, Beijing 100071, People’s Republic of China

**Keywords:** HER2-positive, breast cancer, TKI treatment, antibody drug conjugates, trastuzumab deruxtecan

## Abstract

**Background:**

Both novel anti-human epidermal growth factor receptor 2 (HER2) antibody-drug conjugates (ADCs) and pertuzumab and trastuzumab (HP) combined with chemotherapy(C) regimens are the choice of treatment for HER2 positive metastatic breast cancer (MBC) after tyrosine kinase inhibitors (TKIs). Our team’s previous research has shown significant therapeutic effects of novel anti-HER2 ADCs in patients with TKIs treatment failure. Unfortunately, there is currently no data available to compare novel anti-HER2 ADCs with HP combined with chemotherapy regimens. This study was conducted to compare the efficacy and safety of novel anti-HER2 ADCs with that of the HP combined with chemotherapy regimen in patients for whom TKI treatment failed.

**Materials and methods:**

HER2-positive MBC who used novel anti-HER2 ADCs and HP combined with a chemotherapy regimen from January 2019 to August 2023 were included, and all patients received TKIs. The primary study endpoint was progression-free survival (PFS), while the secondary study endpoints were objective response rate (ORR), clinical benefit rate (CBR), and safety.

**Results:**

A total of 150 patients, of which 83 are in the novel anti-HER2 ADCs group and 67 are in the HP combined with chemotherapy. Among these novel anti-HER2 ADCs, 36 patients received treatment with trastuzumab deruxtecan (T-Dxd), and 47 patients received treatment with other new types of ADCs. The median PFS of the novel anti-HER2 ADCs group and HP combined with the chemotherapy group were 7.0 months and 8.9 months, respectively, with ORR of 51.8% and 26.9%, and CBR of 69.9% and 65.7%, respectively. In subgroup, patients receiving T-Dxd showed improvement in PFS compared to the HP combined with chemotherapy group. The most common grade 3-4 adverse events in the novel anti-HER2 ADCs group and the HP combined with chemotherapy group were neutropenia and gastrointestinal symptoms.

**Conclusions:**

In HER2-positive MBC for whom TKI treatment has failed, novel anti-HER2 ADCs and the HP combined with chemotherapy regimen both showed moderate efficacy and tolerable toxicity. Novel anti-HER2 ADCs are the preferred treatment recommendation for TKI failure patients. Meanwhile, based on the results of this study, the HP combined with chemotherapy regimen may also be an option, especially for patients with low accessibility.

## Teaser

The aim of this study is to compare the efficacy and safety of a novel anti-HER2 antibody drug conjugate combined with HP (pertuzumab and trastuzumab) chemotherapy regimen in patients with TKI treatment failure.

**Implications for practice**

Both novel anti-human epidermal growth factor receptor 2 (HER2) ADC drugs and pertuzumab and trastuzumab (HP) combined with chemotherapy (C) regimens are the choice of treatment for HER2-positive metastatic breast cancer (MBC) after tyrosine kinase inhibitors (TKIs). Our previous research has shown significant therapeutic effects of novel anti HER2 ADC drugs in patients with TKIs treatment failure. Unfortunately, there is currently no data available to compare novel anti-HER2 ADC drugs with HP combined with chemotherapy regimen. This study was conducted to compare the efficacy and safety of novel anti-HER2 ADC drugs with that of the HP combined with chemotherapy regimen in patients for whom TKI treatment failed.

## Introduction

Human epidermal growth factor receptor-2 (HER2)-positive breast cancer driver genes and therapeutic targets have been identified, and anti-HER2 targeted drugs significantly improve the prognosis of patients with HER2-positive breast cancer.^1,2^ For HER2-positive metastatic breast cancer (MBC), with the advent of the PHILA-regimen, the Chinese guidelines have now endorsed a frontline double blockade based on a small-molecule tyrosine kinase inhibitor (TKI) pyrotinib + trastuzumab and chemotherapy.^3^ The results of 2 important phase III studies, PHENIX and PHOEBE, indicate that pyrotinib exhibits significant efficacy in the population who have failed trastuzumab treatment.^4,5^ Based on the above reasons, TKI drugs are increasingly being used in HER2-positive MBCs that have failed treatment with trastuzumab in China.

Antibody-drug conjugates (ADCs) also play an important role in the second-line and subsequent treatment of HER2-positive MBC. The EMLIA study serves as an effective basis to establish the status of T-DM1 as a second-line treatment internationally.^6^ Subsequently, DESTINY-Breast03 trial reshaped the second-line treatment model of the novel anti-HER2 ADC drug T-Dxd (trastuzumab deruxtecan, DS8201) and recommended T-Dxd in major guidelines both domestically and internationally.^7^ In fact, T-DM1 was usually not available in China before, and the actual second-line and third-line are represented by T-Dxd and pertuzumab and trastuzumab (HP) combined with chemotherapy, albeit ADCs have issues with access, sustainability, and insurance coverage here. Still, the long PFS benefit reported in PHILA appears more convincing to be frontline in China, as totally validated in an Asian population, so that the domestic TKI is preferred in trastuzumab-sensitive and resistant cancers.

For patients who have failed TKI treatment, we should adhere to the concept of “continuous anti HER2 treatment” and continue to suppress HER2 driver genes.^8,9^ The Chinese Society of Clinical Oncology (CSCO) Breast Cancer Guideline 2023 recommends HP+C as subsequent regimens after TKI failure for patients who have not been administered pertuzumab before. Such a choice to administrate pertuzumab as a beyond regimen is based on a lower level of evidence.^10,11^ The PHEREXA study used pertuzumab for subsequent treatment in cases of trastuzumab resistance, but the results have not yet demonstrated the benefits of pertuzumab.^11^ Our previous research has shown significant therapeutic effects of novel anti-HER2 ADCs in patients with TKI failure.^12^ However, there is no randomized controlled study comparing the efficacy and safety of the above 2 regimens. Therefore, this study was conducted to compare the efficacy and safety of novel anti-HER2 ADCs with that of the HP combined with chemotherapy regimen in patients for whom TKI treatment failed.

## Methods

### Patient population

This study included patients with HER2-positive MBC treated in the Breast Cancer Ward, Department of Oncology, Chinese People’s Liberation Army General Hospital, from January 2019 to August 2023. The inclusion criteria were as follows: female patients with complete medical records and an Eastern Cooperative Oncology Group (ECOG) score of 0-1; patients with pathology-confirmed HER2-positive MBC, immunohistochemistry- or FISH-confirmed HER2+++, if a metastatic site could not be rebiopsied, the HER2 status was determined using the primary tumor specimen; patients with at least one measurable extracranial lesion or osteolytic and mixed bone metastases and normal heart, liver, kidney, and lung function in accordance with the Response Evaluation Criteria in Solid Tumors (RECIST 1.1); for patients who have previously failed treatment with pyrotinib, treatment failure is defined as interruption of treatment due to disease progression, patient refusal to continue treatment, and other factors (such as adverse events). Follow up with the use of novel anti-HER2 ADCs or HP combined chemotherapy regimen for more than 6 weeks and at least one efficacy evaluation. The exclusion criteria were as follows: patients with symptomatic brain metastases; patients with breast cancer combined with a second primary malignant tumor or serious concomitant diseases.

All data (baseline characteristics, treatment history, efficacy evaluation, and safety data) were collected from the medical records (electronic medical record system) of patients who met the inclusion criteria. The last follow-up was on August 1, 2023.

### Treatment protocols and dose modifications

All patients in the novel anti-HER2 ADCs group were treated with anti-HER2 ADCs, mainly T-Dxd and drugs with a similar mechanism; the drugs were administered in accordance with standard doses: T-Dxd, 3.6 mg/kg, every 3 weeks (q3w); RC48, 2.0 mg/kg, q2w; MRG002, 2.6 mg/kg, q3w; and ARX788, 1.5 mg/kg, q3w. The doses in the HP combined with chemotherapy group were as follows: trastuzumab (initial 8 mg/kg then 6 mg/kg, q3w) and pertuzumab (initial 840 mg then 420 mg, q3w) combined with taxanes (docetaxel 75 mg/m^2^, q3w; and paclitaxel-albumin, 260 mg/m^2^,d1, d8, q3w, vinorelbine, 25 mg/m^2^, d1, d8, q3w, or gemcitabine, 1000 mg/m^2^, d1, d8, q3w). After 6-8 cycles of combined chemotherapeutic drugs, a senior physician adjusted the dose or stopped the chemotherapy drugs based on efficacy and tolerance and considered whether there was a need to switch to endocrine drugs based on the hormone receptor situation.

### Outcomes and assessments

The primary endpoint of the study was PFS, defined as the time from the first use of the study drug until confirmed disease progression (as assessed by CT/MRI) or death from any cause. Secondary endpoints included the objective response rate (ORR), clinical benefit rate (CBR), and safety. The ORR was defined as the proportion of patients with complete remission (CR) and partial remission (PR). The CBR was defined as the proportion of patients with CR, PR, and SD ≥ 6 months. The curative effect of target lesions was evaluated every 2 cycles using RECIST 1.1. Adverse events during the whole course of medication were traced and graded in accordance with the common terminology criteria for adverse events (CTCAE) version 4.0.

### Statistical analysis

Statistical analysis was performed using SPSS 19.0. Continuous variables were analyzed with the *t* test or Wilcoxon rank-sum test, and the *χ*^2^ test or Fisher’s exact probability test was used to compare categorical variables as well as differences in the ORR and CBR. The Kaplan-Meier method was used to analyze PFS and plot survival curves, and the log-rank test was used to compare curves. Hazard ratios (HRs) with 95% CIs were calculated using a Cox proportional hazards model. To explore the prognostic impact of predetermined baseline information on PFS, subgroup analyses were performed using Cox proportional risk models, and the results are shown in forest plots. Forest plots were created in GraphPad Prism version 7.0. All tests were 2-sided, and *P* < .05 was considered statistically significant.

## Results

### Clinical characteristics

**Figure 1.**
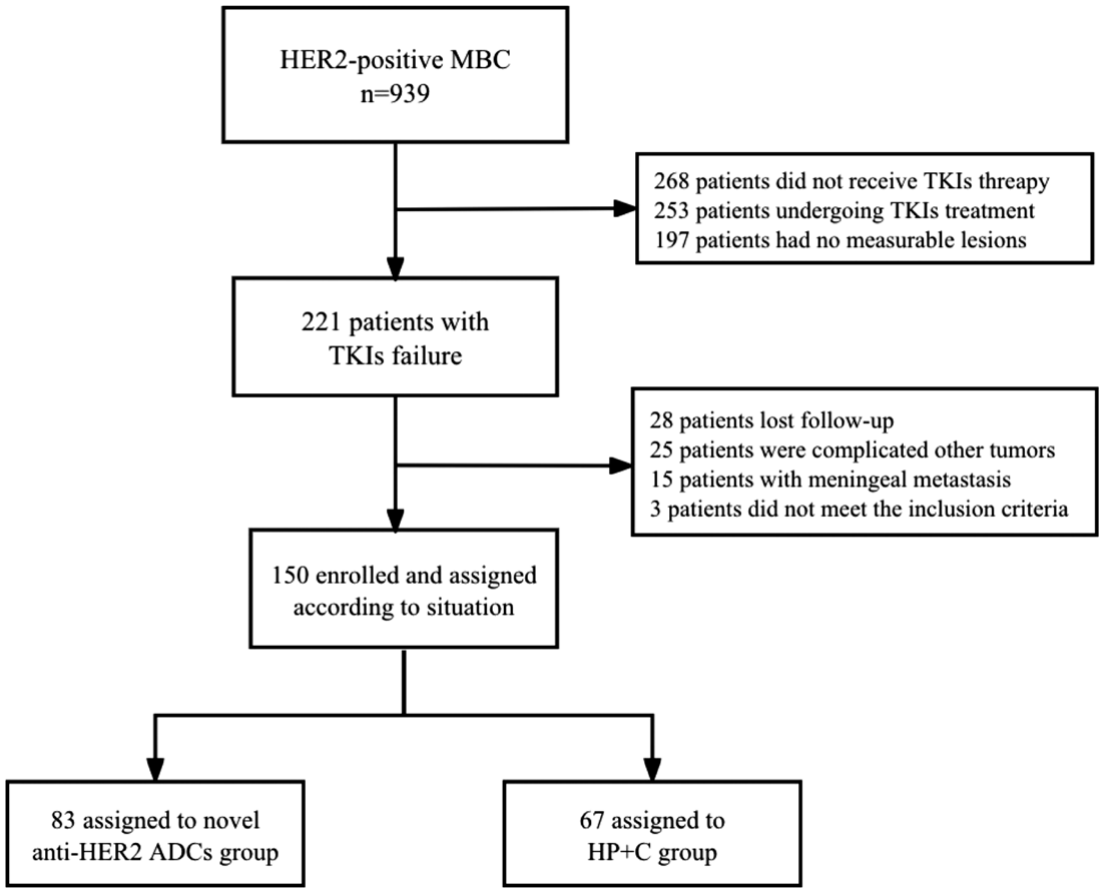
Selection of 150 women with HER2-positive metastatic breast cancer treated with novel anti-HER2 ADCs and HP+C. HER2, human epidermal growth factor receptor 2; ADCs, antibody-drug conjugates; HP+C, pertuzumab + trastuzumab + chemotherapy; TKI, tyrosine kinase inhibitor.

From January 2019 to August 2023, a total of 150 patients who met the inclusion criteria were included in the study. The research flow chart is shown in Figure 1. The median age at diagnosis was 48 years (25-89 years). A total of 83 patients in the novel anti-HER2 ADCs group received the following treatments: T-Dxd (36 patients, 43.4%), MRG002 (24 patients, 28.9%), ARX788 (13 patients, 15.7%), and RC48 (10 patients, 12.0%). In the HP combined with chemotherapy group, 67 patients received HP combined with chemotherapy drugs: 47 patients (70.1%) received taxanes, 11 patients (16.4%) received vinorelbine, and 9 patients (13.4%) received gemcitabine. The baseline characteristics of the 2 groups were basically similar, and the proportions of patients with liver, lung, and brain metastases were basically well balanced. However, there were significantly more patients with multiple metastases (metastases ≥ 3) in the novel anti-HER2 ADCs group than the HP combined with chemotherapy group (62.7% vs 31.3%, *P* < .001). More than half of the patients in the novel anti-HER2 ADCs group had received ≥ 3 lines of anti-HER2 treatment; the percentage of patients with ≥ 3 lines of anti-HER2 treatment was significantly higher in the novel anti-HER2 ADCs group than the HP combined with chemotherapy group (54.2% vs 32.8%, *P* = .008). All patients had received trastuzumab and pyrotinib. The benefits of previous treatment were basically similar, except that 62 patients (74.7%) in the novel anti-HER2 ADCs group had previously received pertuzumab therapy (Table 1).

**Table 1.** Demographic and baseline clinical characteristics of 150 women with HER2-positive MBC treated with novel anti-HER2 ADCs and HP+C.

Characteristic	Novel anti-HER2 ADCs (*N* = 83)	HP+C (*N* = 67)	*P-*value
Age at diagnosis, years			.787
Median (range)	48 (25-89)	48 (25-72)	
<50	44 (53.0)	37 (55.2)	
≥50	39 (47.0)	30 (44.8)	
Hormone receptor			.981
Negative	37 (44.6)	30 (44.8)	
Positive	46 (55.4)	37 (55.2)	
Clinical stages at diagnosis			.225
I	9 (10.8)	8 (11.9)	
II	31 (37.3)	30 (44.8)	
III	27 (32.5)	24 (35.8)	
IV	16 (19.4)	5 (7.5)	
Number of metastatic lesions			<.001
< 3	31 (37.3)	46 (68.7)	
≥ 3	52 (62.7)	21 (31.3)	
Metastatic lesion			
Liver	42 (50.6)	31 (46.3)	.598
Lung	48 (57.8)	36 (53.7)	.615
Brain	20 (24.1)	22 (32.8)	.236
Bone	44 (53.0)	36 (53.7)	.930
Previous anti-HER2 therapy			
Trastuzumab	83 (100.0)	67 (100.0)	–
Trastuzumab + Pertuzumab	62 (74.7)	0 (0)	<.001
Pyrotinib	83 (100.00)	67 (100.0)	–
Lapatinib	18 (21.7)	29 (43.3)	<.001
Number of previous lines of anti-HER2 therapy			.008
≤ 3	38 (45.8)	45 (67.2)	
> 3	45 (54.2)	22(32.8)	
Benefits of previous treatment			
Trastuzumab benefits	63 (75.9)	50 (74.6)	.857
TKI benefits	58 (69.9)	52(77.6)	.287

Abbreviations: ADCs, antibody-drug conjugates; HER2, human epidermal growth factor receptor 2; HP+C, pertuzumab + trastuzumab + chemotherapy; TKI, tyrosine kinase inhibitor.

### Efficacy

As of August 2023, the median follow-up time was 6 months (2-25.6 months). In the anti-HER2 ADCs group, 24 patients (28.9%) were on treatment, medication was discontinued in 58 (69.9%) patients due to disease progression, and medication was discontinued in 1 (1.2%) patient due to interstitial disease. In the HP combined with chemotherapy group, 5 patients (7.5%) were on medication, and medication was discontinued in 62 patients (92.5%) due to disease progression. No patients in either the novel anti-HER2 ADCs group or the HP combined with chemotherapy group achieved CR; the PR rates were 51.8% and 26.9%, respectively, and the SD rates were 43.4% and 64.2%, respectively. There was a statistically significant difference between the novel anti-HER2 ADC group and the HP combined with chemotherapy group in the ORR (51.8% vs 26.9%, *P* = .002) and no significant difference in the CBR (69.9% vs. 65.7%, *P* = .583) (Table 2).

**Table 2.** Comparison of efficacy between novel anti-HER2 ADCs and HP+C.

Type of response no. (%)	Novel anti-HER2 ADCs (*n* = 83)	^HP+C^ (*n* = 67)	*P-*value
CR	0	0	
PR	43 (51.8)	18 (26.9)	
SD	36 (43.4)	43 (64.2)	
SD ≥6	17 (20.5)	26 (38.8)	
PD	4 (4.8)	6 (9.0)	
ORR	43 (51.8)	18 (26.9)	.002
CBR	58 (69.9)	44 (65.7)	.583

Abbreviations: ADCs, antibody-drug conjugates; CBR, clinical benefit rate; CR, complete response; HER2, human epidermal growth factor receptor 2; HP+C, pertuzumab + trastuzumab + chemotherapy; ORR, objective response rate; PD, progressive disease; PR, partial response; SD, stable disease.

**Figure 2.**
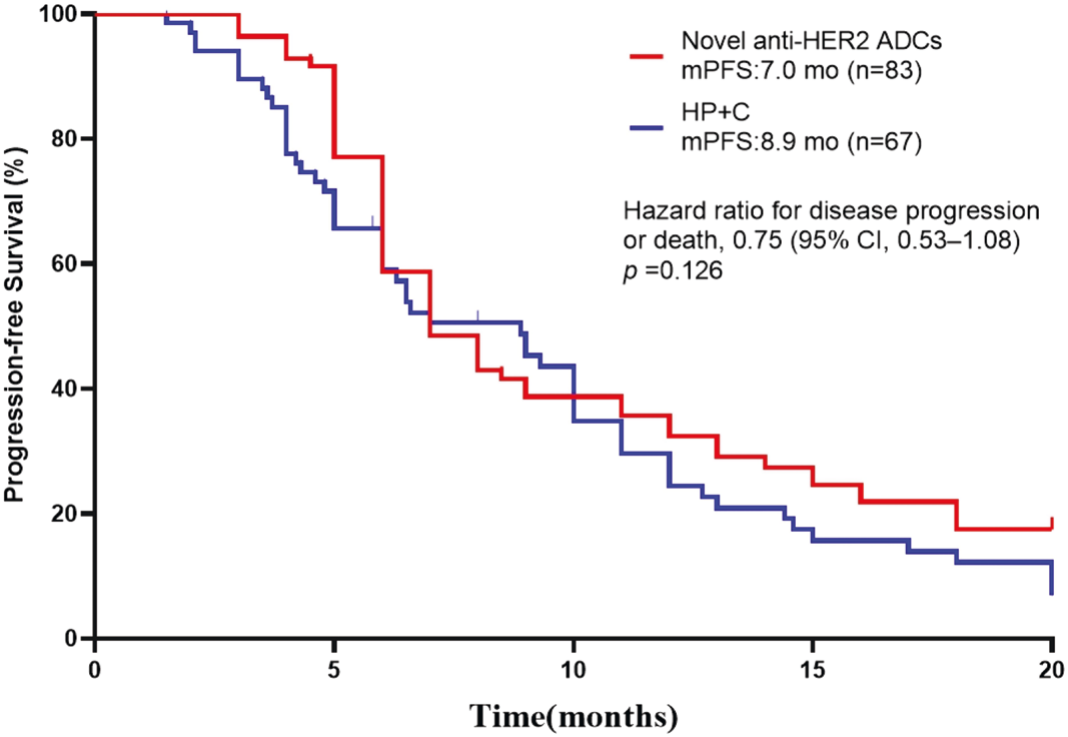
Kaplan-Meier analysis of progression-free survival (PFS) for all patients treated with Novel anti-HER2 ADCs and HP+C. Abbreviations: ADCs, antibody-drug conjugates; CI, confidence interval; HER2, human epidermal growth factor receptor 2; HP+C, pertuzumab + trastuzumab + chemotherapy; mo, month

**Figure 3.**
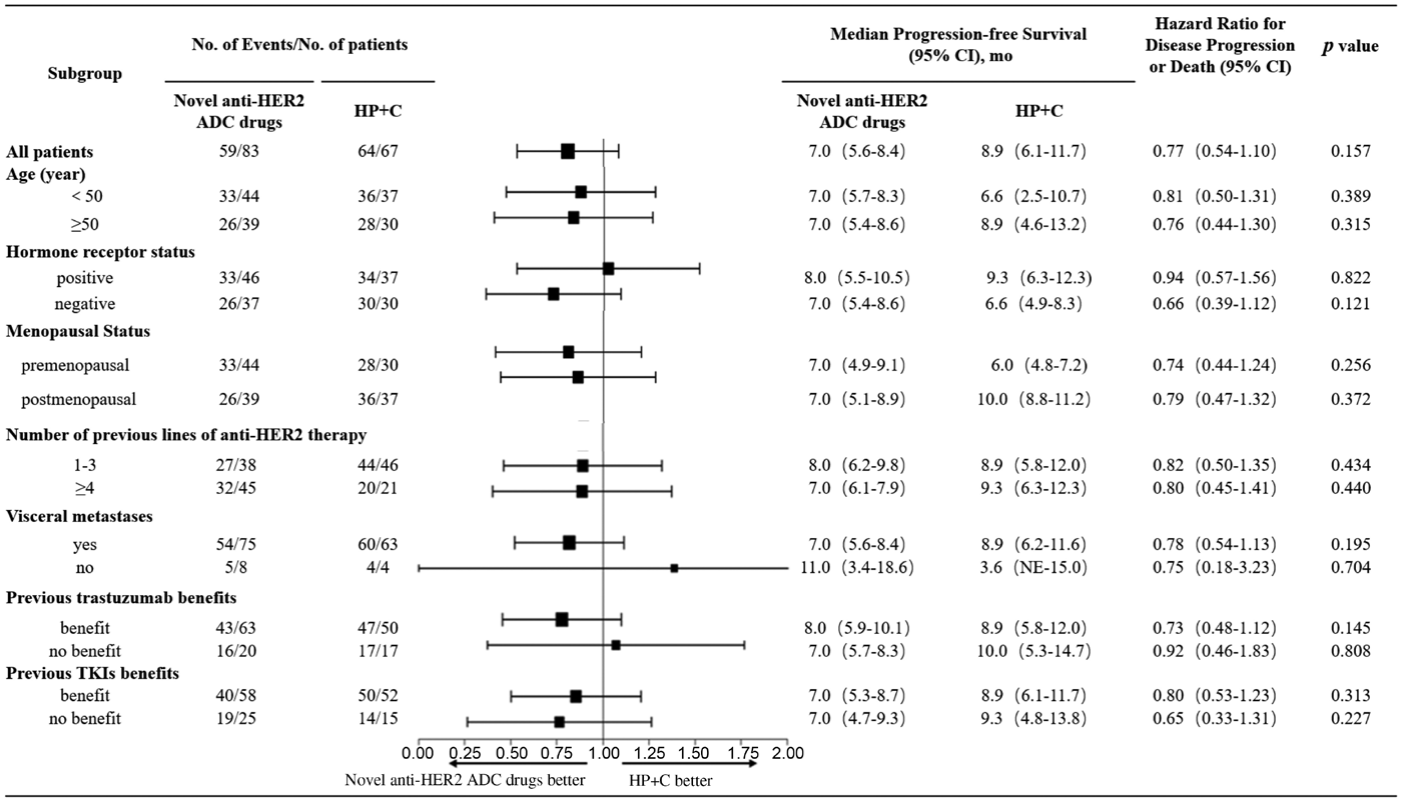
Subgroup analysis of progression-free survival (PFS). Abbreviations: ADCs, antibody-drug conjugates; CI, confidence interval; HER2, human epidermal growth factor receptor 2; HP+C, pertuzumab + trastuzumab + chemotherapy; mo, month; TKI, tyrosine kinase inhibitor.

The median PFS in the novel anti-HER2 ADC group was 7.0 months, and that in the HP group was 8.9 months; there was no significant difference between the 2 groups (HR = 0.75, 95% CI, 0.53-1.08, *P* = .126, Figure 2). Forest plots indicated no differences in PFS among all subgroups, including age, hormone receptor status, menopausal status, previous number of lines of anti-HER2 therapy, visceral metastasis, previous trastuzumab benefit, and previous TKI benefit (Figure 3).

**Figure 4.**
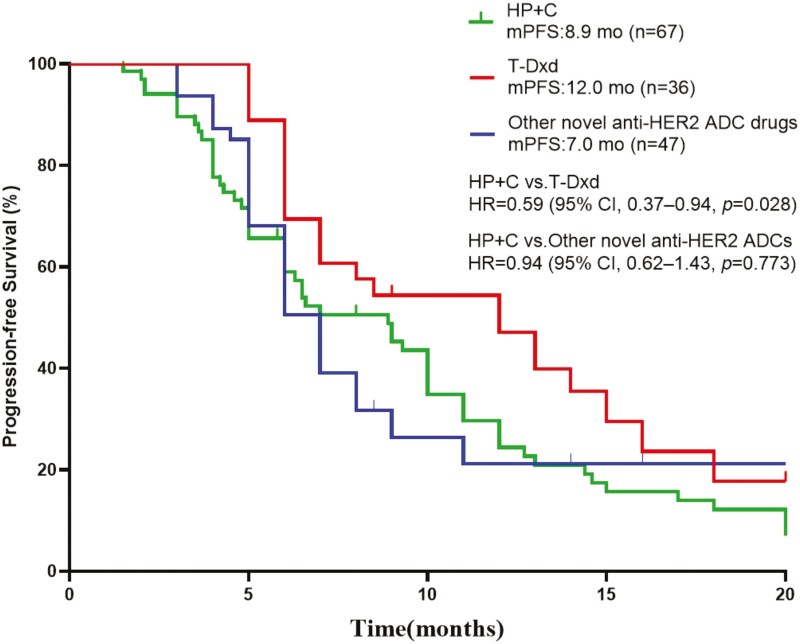
Kaplan-Meier analysis of progression-free survival (PFS) for all patients treated with HP+C, T-Dxd and other novel anti-HER2 ADCs. Abbreviations: ADCs, antibody-drug conjugates; CI, confidence interval; HER2, human epidermal growth factor receptor 2; HP+C, pertuzumab + trastuzumab + chemotherapy; mo, month.

In further subgroup analyses, the patients were divided into 3 groups, with 36 patients in the T-Dxd group, 67 patients in the HP combined with chemotherapy group, and 47 patients in the other novel anti-HER2 ADCs group. Survival analysis of the 3 groups of patients was performed. The median PFS in the HP combined chemotherapy group, in the T-Dxd group, in the other novel anti-HER2 ADCs group was 8.9 months, 12.0 months, 7.0 months, respectively. The difference between the HP combined chemotherapy group and the T-Dxd group is statistically significant (HR = 0.59, 95% CI, 0.37-0.94, *P* = .028, Figure 4).

### Safety

The safety data of the 2 groups are shown in Table 3. Compared with that in the HP combined with chemotherapy group, the incidence of grade 3-4 adverse events in the novel anti-HER2 ADCs group was relatively higher, especially for nausea (10.8%), vomiting (10.8%), leukopenia (9.6%), and diarrhea (8.4%). Five patients in the novel anti-HER2 ADCs group developed interstitial pneumonia: 1 patient was classified as grade 4 and withdrew from the clinical study. The grade 3-4 adverse reactions in the HP combined with chemotherapy group mainly included nausea (17.9%), neutropenia (16.4%), and vomiting (14.9%). There were no grade 5 adverse events in the 2 groups. All adverse events resolved or improved after symptomatic treatment, and no patient died due to adverse events. Overall, the safety of both groups of patients is manageable.

**Table 3.** Treatment-related adverse events in the 2 groups (*n*, %).

Adverse event	Novel anti-HER2 ADCs (*n* = 83)		HP+C (*n* = 67)	
	Any grade	Grades 3-4	Any grade	Grades 3-4
Leukopenia	29 (34.9)	8 (9.6)	27 (40.3)	5 (7.5)
Neutropenia	24 (28.9)	7 (8.4)	23 (34.3)	11 (16.4)
Anemia	32 (38.6)	6(7.2)	18 (26.9)	5 (7.5)
Thrombocytopenia	17 (20.5)	5 (6.0)	10 (14.9)	3 (4.5)
Elevated transaminase	16 (19.3)	3 (3.6)	19 (28.4)	4 (6.0)
Diarrhea	18 (21.7)	7 (8.4)	47 (70.1)	0
Constipation	15 (18.1)	1 (1.2)	15 (22.4)	0
Nausea	58 (69.9)	9 (10.8)	50 (74.6)	12 (17.9)
Vomiting	37 (44.6)	9 (10.8)	29 (43.3)	10 (14.9)
Fatigue	50 (60.2)	5 (6.0)	48 (71.6)	4 (6.0)
Decreased appetite	33 (39.8)	0	28 (41.8)	0
Peripheral neuropathy	21 (25.3)	5 (6.0)	28 (41.8)	3 (4.5)
Interstitial lung disease	5 (6.0)	1 (1.2)	0	0
LVEF (< 50% or ≥ 15% reduction from baseline)	0	0	1 (1.5)	0

Abbreviations: ADCs, antibody-drug conjugates; HER2, human epidermal growth factor receptor 2; HP+C, pertuzumab + trastuzumab + chemotherapy; LVEF, left ventricular ejection fraction.

## Discussion

The development of new drugs provides more options for the later-line treatment of HER2-positive MBC. For those in whom TKI treatment has failed, finding the best treatment regimen is crucial to prognosis. This is the first real-world study to date that compared novel anti-HER2 ADCs and HP combined chemotherapy regimens in patients after TKI treatment failure. The results showed that there was a significant difference in the ORR between the novel anti-HER2 ADCs and the HP combined with chemotherapy group and that there was no statistically significant difference in the median PFS or the CBR between the 2 groups. Subgroup analysis showed that there was no difference in PFS between the 2 groups regardless of age, hormone receptor status, menopausal status, number of anti-HER2 lines of treatment, visceral metastasis, trastuzumab treatment benefit, and TKI treatment benefit. The safety data indicated that tolerability was acceptable in the 2 groups of patients, with no adverse events other than known toxicity and no deaths due to toxicity.

In this study, the median PFS in the novel anti-HER2 ADCs group and in the HP combined with chemotherapy regimen group was 7.0 months and 8.9 months, respectively (*P* = .126). Previous randomized controlled studies showed that the median PFS in patients who received the novel anti-HER2 ADCs T-Dxd ranged from 16.4 to 25.1 months and that the ORR ranged from 60.9% to 79.7%^.7,13^ Compared with randomized controlled studies, the median PFS in the novel anti-HER2 ADCs group in this study was significantly lower. There are 2 main reasons for this difference in efficacy. First, randomized controlled studies include a strictly selected population, whereas real-world patients have more concurrent underlying diseases and heavier tumor loads, and thus, the observed efficacy of the same drug is often inferior to that in randomized controlled studies.^14,15^ Notably, in this study, 89% of patients in the novel anti-HER2 ADCs group had visceral metastases, higher than the 70% of patients in the DESTINY-Breast03 study. Additionally, the patients’ baseline characteristics and disease characteristics and previous treatment conditions may also be a reason for the disparity in efficacy.^16,17^ Second, T-Dxd only accounted for 40% of the treatments in the novel anti-HER2 ADCs group, and although other novel anti-HER2 ADC drugs have mechanisms similar to that of T-Dxd, some are still in the clinical trial stage, and their efficacy is not completely clear. Therefore, we divided the novel anti-HER2 ADCs group into T-Dxd group and other novel anti-HER2 ADCs group, and compared them with the HP combined with chemotherapy group to form 3 sets of data. The results showed significant statistical differences between the T-Dxd group and the HP combined with chemotherapy group, a finding that may be related to the small sample size and lack of interaction analysis.

The HP combined with chemotherapy regimen is currently the standard first-line treatment for HER2-positive metastatic breast cancer. The CLEOPATRA study^18^ showed that the HP combined with chemotherapy regimen increased the median PFS of patients from 12.4 months to 18.7 months, with a median overall survival (OS) of 57.1 months^.19^ The PUFFIN study confirmed the same benefit in a Chinese population^.20^ However, due to the fact that pertuzumab was only launched in China in 2019, many patients only use trastuzumab combined with chemotherapy in the first-line treatment stage, and there are fewer data on the HP combined with chemotherapy regimen in the later-line treatment stage,^10^ The rare phase III randomized controlled study PHEREXA trial results on the use of pertuzumab after trastuzumab failure were only negative.^11^ Previous studies have confirmed that the efficacy of a drug will decrease as the number of lines of treatment increases.^21,22^ In this study, the median PFS in the HP combined with chemotherapy group was 8.9 months, far shorter than the first-line treatment efficacy reported in the CLEOPATRA study and the PUFFIN study.^19,20^ However, the efficacy was similar to that of the novel anti-HER2 ADCs group. From the perspective of drug availability and medical insurance reimbursement policies, patients in China are more likely to receive the HP combined with chemotherapy regimen.

Subgroup analysis showed that regardless of whether previous trastuzumab and TKI treatments were beneficial, there was no significant difference between novel anti-HER2 ADCs and the HP combined with chemotherapy regimen. Previous studies have reported a median PFS of 3.4 months with trastuzumab alone in a population for whom TKI therapy failed.^22^ In this study, the improvement in the median PFS in the HP combined with the chemotherapy regimen group may be related to the effect of pertuzumab. Trastuzumab can block ligand-independent HER2-HER3 signaling^23^ but cannot block ligand-dependent HER2-HER3 signaling. In contrast, pertuzumab blocks ligand-gated HER2-HER3 signaling,^24^ and the synergistic and complementary mechanism of the 2 can comprehensively block HER2 channel signal transduction.^25^ Based on the results of this study, the HP combined with chemotherapy regimen can prolong PFS more than the single-target therapy regimen.^22^ Currently, we are conducting translational research to select a suitable population for the HP combined with chemotherapy regimen.

The safety data further showed the efficacy advantage of the HP combined with chemotherapy regimen. The adverse events of the 2 groups of patients mainly manifested as hematology and gastrointestinal toxicity, events similar to those described previously.^7,20^ There were significantly more grade 3-4 adverse events in the novel anti-HER2 ADCs group than in the HP combined with chemotherapy group, even exceeding the values reported in the DESTINY-Breast03 study.^7^ These results may be due to the combined effect of many factors, including drug load, tumor load, previous medication, and number of lines of treatment. Additionally, 5 patients in the novel anti-HER2 ADC group developed interstitial lung disease, an effect that has also been reported in previous studies. One patient had grade 4 toxicity, and the symptoms resolved after drug withdrawal and symptomatic treatment. Such effects increase resistance to the use of novel anti-HER2 ADCs. The monitoring, diagnosis and management of interstitial pneumonia deserve further exploration.^26^ In the HP combined with chemotherapy group, hematological toxicity, and abnormal liver function were considered related to the chemotherapy drugs.^20^ During the treatment period, no patient developed clinical symptoms related to cardiotoxicity.

This study has certain limitations, such as a small sample size, selection bias, and lack of randomization between groups. The retrospective nature of the study also limits the quantification of the effects of potential confounding variables. Despite these limitations, this study still holds significant reference value for treatment options for HER2-positive metastatic breast cancer patients after TKI treatment failure.

## Conclusions

In summary, in HER2-positive MBC for whom TKI treatment has failed, the novel anti-HER2 ADCs and the dual anti-HER2 antibody combined with chemotherapy regimen both showed moderate efficacy and tolerable toxicity. Novel anti-HER2 ADCs are the preferred treatment recommendation for TKI failure patients. Meanwhile, based on the results of this study, the dual anti-HER2 antibody combined with chemotherapy regimen may also be an option, especially for patients with low accessibility.

## Data Availability

The data underlying this article will be shared on reasonable request to the corresponding author.
